# *De novo* transcriptome for *Chiloscyllium griseum*, a long-tail carpet shark of the Indian waters

**DOI:** 10.1038/s41597-024-03093-7

**Published:** 2024-03-09

**Authors:** Pooja Harshan, Sandhya Sukumaran, A. Gopalakrishnan

**Affiliations:** 1 Marine Biotechnology, Fish Nutrition and Health Division, ICAR-Central Marine Fisheries Research Institute, Ernakulam North P.O., Kochi, Kerala 682018 India; 2 Cochin University of Science and Technology, South Kalamassery, Ernakulam, Kerala 682022 India

**Keywords:** RNA sequencing, Next-generation sequencing

## Abstract

Sharks have thrived in the oceans for 400 million years, experienced five extinctions and evolved into today’s apex predators. However, enormous genome size, poor karyotyping and limited tissue sampling options are the bottlenecks in shark research. Sharks of the family Orectolobiformes act as model species in transcriptome research with exceptionally high reproductive fecundity, catch prominence and oviparity. The present study illustrates a *de novo* transcriptome for an adult grey bamboo shark, *Chiloscyllium griseum* (Chondrichthyes; Hemiscyllidae) using paired-end RNA sequencing. Around 150 million short Illumina reads were obtained from five different tissues and assembled using the Trinity assembler. 70,647 hits on Uniprot by BLASTX was obtained after the transcriptome annotation. The data generated serve as a basis for transcriptome-based population genetic studies and open up new avenues in the field of comparative transcriptomics and conservation biology.

## Background & Summary

The evolution of sharks stretches back from humble proportions up to 100 million years to today’s apex predators of the ocean. The fact that many modern sharks evolved millions of years ago and have remained consistent throughout that time demonstrates how competent and well-integrated these creatures are in their ecological niches. Over millions of years of evolution, today’s Selachii have established some of the most sophisticated hunting systems ever known^1^. Sharks’ success as predators is largely due to their highly developed sensory systems^2^. Since sharks are just incredibly hardy, it’s more likely that their wonderful diversity is key to their success. No wonder they have ruled the ocean for hundreds of millions of years.

Selachians are often described as organisms with prolonged reproductive cycles, enormous body size, gradual growth rate, delayed sexual maturity, low reproductive fertility, and a relatively long lifespan, making their conservation in the laboratory difficult^3,4^. All of these factors have been the major bottlenecks in molecular biology research on cartilaginous fish. Researchers were keen to work on other model organisms with smaller body sizes and short generation cycles such as zebrafish, nematodes, fruit flies and mice, which took biological research to higher dimensions^5^. However, recent studies suggest that elasmobranch non-coding sequences share homology with humans, making them easily comparable, rather than those of teleosts and humans^6–8^. This comparison has been hypothesized to be due to the finely tuned and lengthy molecular clock in cartilaginous fish^3,9,10^. Molecular data encoding biological information in elasmobranchs is scarce in a limited number of species, and transcriptome data from this important group could encourage comparative studies.

The development of gnathostomes (mandibular vertebrates) is characterized by various physiological and morphological adaptations such as articulated jaws, paired fins, and immunoglobulin-based adaptive immunity^9^. The immune system of cartilaginous fish is very similar to that of mammals with regard to immunoglobulins (Igs), T cell receptors (TCRs), recombination activation gene proteins (RAG) and major histocompatibility complex molecules (MHC). However, immunogenetic studies in cartilaginous fish are hampered by bottlenecks in sequencing immune genes and a lack of molecular research tools. Decoding the entire genomic information of the great white shark, *Carcharodon carcharias* has revolutionized the field of marine research and has provided evidence for a variety of genetic alterations^11^. Genome stability is the most important factor that keeps sharks in the premier class of vertebrates, giving them superior abilities to fight deadly diseases like cancer and other age-related diseases compared to humans. Shark genomes also shed light on genes’ evolutionary adaptations to wound-healing traits.

Recently, elasmobranch transcriptome data are increasingly used to estimate population size and evolutionary divergence in population genetics studies^12,13^. Also, Evolutionary Distinctness (ED), which is a measure of a species’ uniqueness, considers a molecular phylogenetics-based score that can be used to implement conservation prioritization^14,15^. This molecular information would be useful in formulating better conservation policies for sharks. Recent developments in shark studies include improved genome assembly of the whale shark and *de novo* whole-genome assembly of the clouded catshark and brown- banded bamboo shark. Many projects linked to the global genome sequencing initiative Earth Biogenome Project (EBP)^16^ are sequencing the entire genomes of more diverse shark and ray species. These projects include the Vertebrate Genome Project (VGP)^17^, Fish 10K^18^, Darwin Tree of Life (https://www.darwintreeoflife.org/), and Squalomix (https://github.com/Squalomix/info), an omics project led by Nishimura *et al*.^19^, specifically focused on cartilaginous fish. The results of these initiatives, along with the development of laboratory solutions, will increase the currently restricted viability of long-term studies on cartilaginous fishes in the field of developmental Biology.

**Fig. 1:**
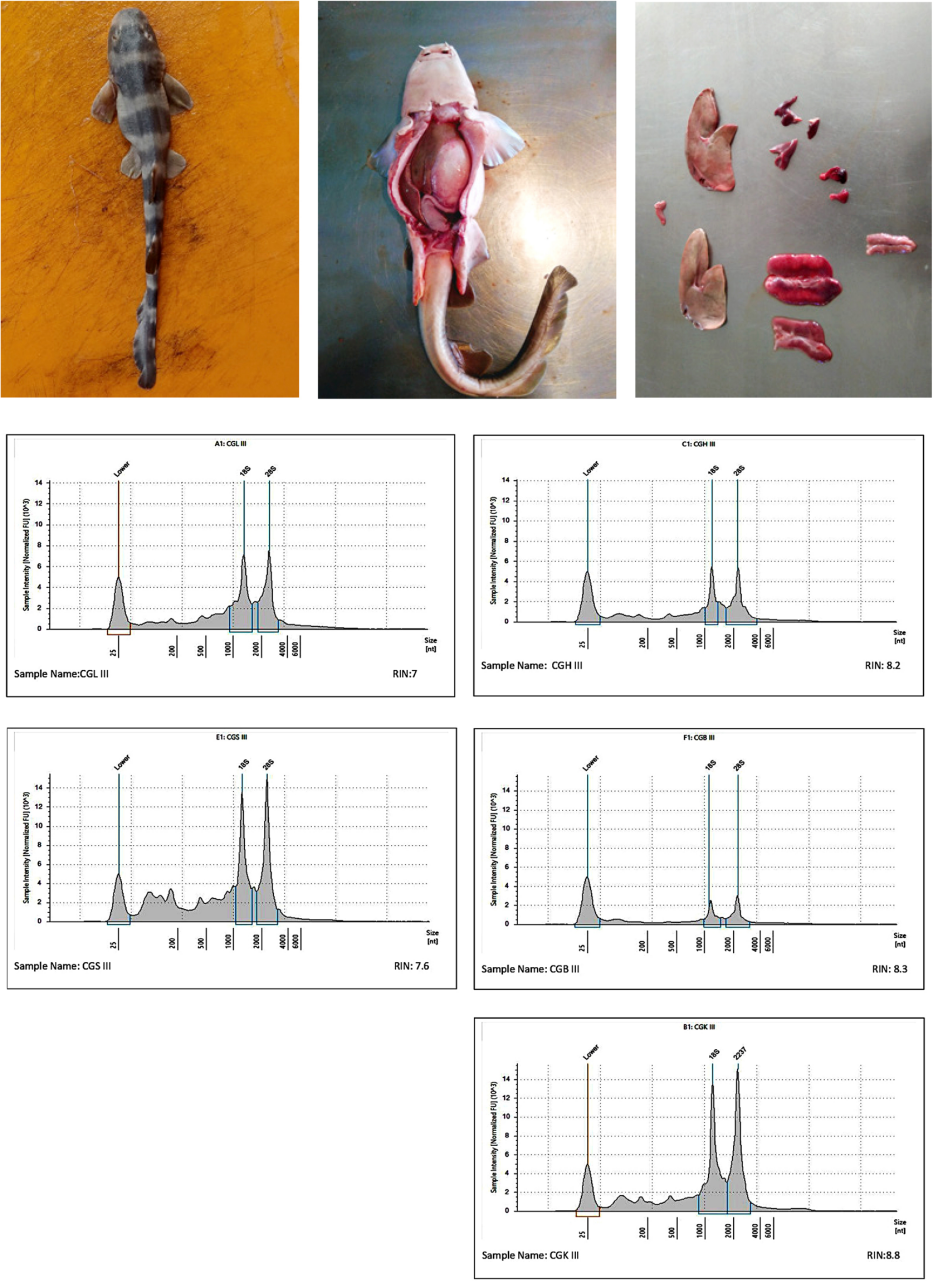
The Grey bamboo shark and sample preparation. (**a**) Juvenile grey bamboo shark. (**b**) Live bamboo shark before dissection. (**c**) Dissected tissues of grey bamboo shark. RNA length distribution analysis of liver (**d**), heart (**e**), spleen (**f**), brain (**g**) and kidney (**h**) tissues on the bioanalyzer 2100 respectively.

In the present study, we report transcriptome data from the grey bamboo shark (*Chiloscyllium griseum*; Fig. 1a). The grey bamboo shark is an oviparous species of elasmobranch commonly found in the Indo-West Pacific from India to Australia^20^. This belongs to the order Orectolobiformes and family Hemiscyllidae and consists of two valid genera with seventeen species and a moderately high ED score^21^. The grey bamboo shark is currently listed as ‘Vulnerable’ in the IUCN Red List 2020^22^. The grey bamboo shark reference transcriptome would thus be a potential molecular resource for the characterization of species in this genus in the foreseeable future. An adult female grey bamboo shark was collected at Neendakara Fishing Port. 482,871 assembled contigs were generated from paired-end RNA libraries through Illumina HiSeq technology. From the assembled transcripts, approximately 70,647 protein-coding sequences were predicted.

## Methods

### Generation of datasets

The wild specimens of *Chiloscyllium griseum* (Grey bamboo shark) were collected from the Neendakara Fishery Harbour, Kollam, Kerala (8°56′18.32″N 76°32′33.78″ E) using fish gears such as bottom set gillnets and trawl nets and crafts like outboard fiber boats and trawlers. Species identity was confirmed by both morphological characters and molecular analyzes comprising of DNA barcoding. The sequence entries confirming the species, ‘*Chiloscyllium griseum’* from DNA barcoding were deposited in the NCBI Genbank (PP059596-PP059597). The shark sample used in the present study was carefully handled following the guidelines for the care and use of fish in research by De Tolla *et al*.^23^. The protocols for animal experimentation were set up in compliance with the standards approved by the Institutional Animal Ethical Committee of the ICAR Central Marine Fisheries Research Institute (CMFRI), Kochi. These methods were also testified abiding ARRIVE guidelines (http://arriveguidelines.org). Around five sharks (one female adult and four male juveniles) were maintained at a temperature of 29 °C, 7.5–8.5 pH, 3–6 mg/L dissolved oxygen (DO) and 34–35 ppt salinity for 14 days in a 1000 L tank of the aquarium facility under the hatchery, ICAR CMFRI, Kochi. An adult female grey bamboo shark weighing 905 g and a tail length (TL) of 62 cm was dissected into heart, spleen, brain, kidney and liver (Fig. 1b,c) and flash frozen with liquid nitrogen and kept at −80 °C for RNA extraction. RNA extraction from each of the tissue samples were carried out using RNeasy^®^ Plus Mini kit (QIAGEN, Cat. No. 74134). Genomic DNA (gDNA) present was expelled using gDNA Eliminator columns provided in this kit. For Quality check, Qubit 4 Fluorometer (Invitrogen), NanoDrop One Spectrophotometer (ThermoScientific, USA) and Agilent 2200 TapeStation were used to assess the RNA integrity (RIN) value which generated a score of greater than or equal to 7 for all the samples (Fig. 1d–h) indicating that superior quality RNA was being used for library preparation. As a substratum for RNA-seq, 0.5 μg of RNA from each of the five tissues were extracted from each of the five tissues to create unambiguous RNA libraries or cDNA libraries using TruSeq RNA sample preparation kit v2low-throughput protocol (Illumina, Cat. No. RS-122-2001 and/or RS-122-2002) following manufacturer’s guidelines. Assessment on the quality of cDNA library generated was made with the help of 2100 bioanalyzer (Agilent technologies, Part. No. G2939BA), concentration measured using library quantification kit (KAPA Biosystems, Cat. No. KK4824) and sequenced on HiSeq X10 platform (Illumina) operated by HiSeq control software v.3.5.0. Quality control of the obtained fastq file of both the forward and the reverse strand of the pooledtranscriptome library was executed using FASTQC v0.11.9. Finally, pooled transcriptome sequence reads from each tissue was made available in the public domain with a specific accession. The generated transcriptome data metrics is shown in Table 1.

**Table 1:** List of raw reads.

Organism	sample	Read orientation	protocols	Number of reads obtained	Total number of reads (R1 + R2)	Biosample	Raw data accession (SRA)
*Chiloscyllium griseum*	Pooled RNA of liver, heart, spleen, kidney, brain from female adult shark	R1	RNA isolation, paired-end illumina sequencing	91,043,535	182,087,070	SAMN17193099^30^	SRR15990417^30^
		R2	RNA isolation, paired-end illumina sequencing	91,043,535			

### Data processing

**Fig. 2:**
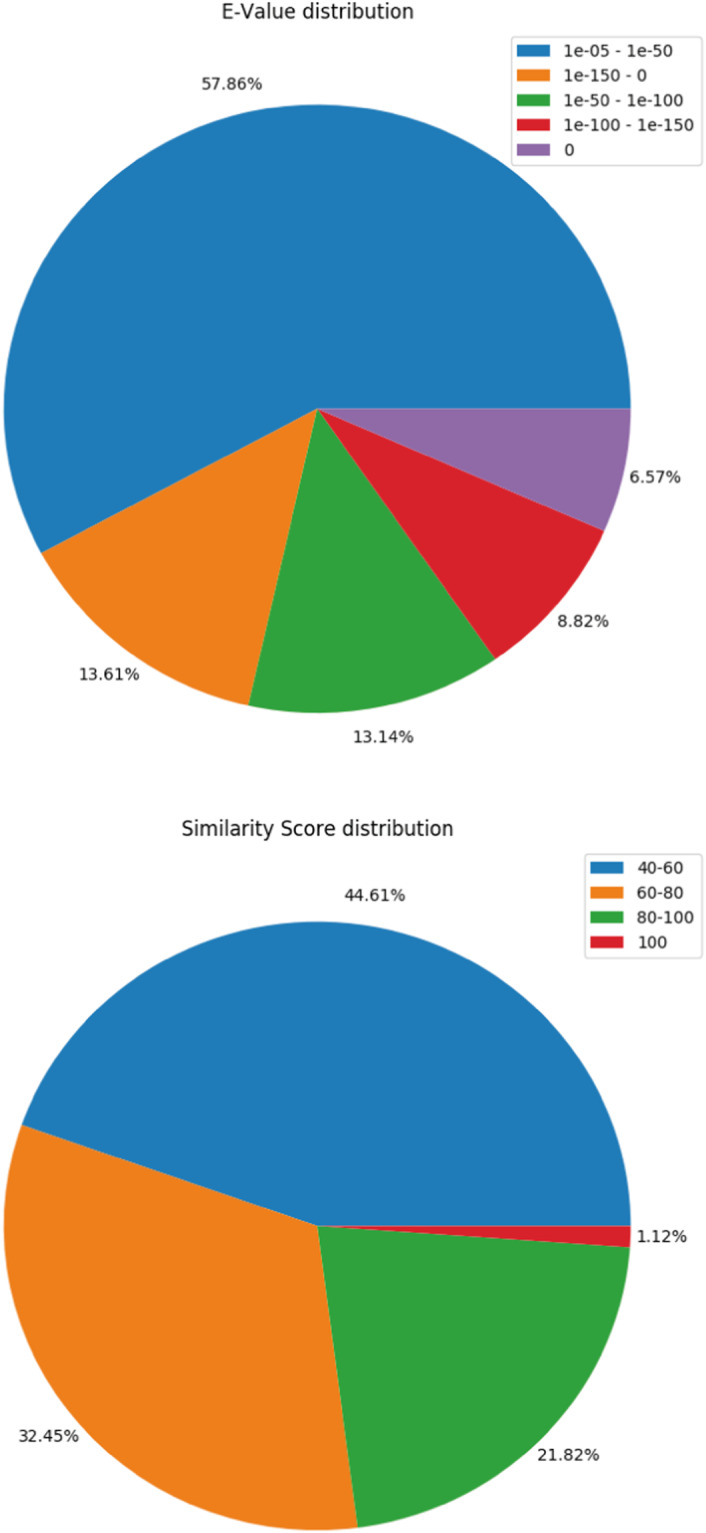
BLASTX summary. (**a**) E-value distribution of BLASTX hits. (**b**) similarity score distribution of the BLASTX hits.

In this dataset, we present the *de novo* reference transcriptome of *Chiloscyllium griseum* (grey bamboo shark), a long-tail carpet shark of the Indian waters. The total sequencing coverage of the pooled sample was in the order of 180 million reads obtained from both the forward (R1) and the reverse (R2) strands. These statistics are provided in Table 1. A reference transcriptome was created through NGS shotgun assembly to retrieve the transcripts from the entire samples with a corresponding minimum length in the range of 200–250 nucleotides. The total number of assembled pair end (PE) reads with maximum quality retrieved was 150,032,276. A sequence trimming pipeline, Trim-galore (toolshed.g2.bx.psu.edu/repos/bgruening/trim_galore/trim_galore version 0.6.7 + galaxy0; parameters:–paired –phred33 -e 0.1 -q 30), low-quality data sets and adapters were eliminated from the dataset. The cleaned reads were further subjected to assembly in a Trinity^24,25^ assembler to yield 4,82,871 contigs/assembled transcripts with a mean GC content of 41.6% and the longest transcript length of 44,554 as directed in Table 2. Similar sequences were clustered using CD-HIT-EST to remove redundant sequences. The clustered transcripts were further filtered using TransDecoder^25^. The assembled transcripts were annotated using an in-house pipeline comprising of three major steps. These are,

- Matching with a Uniprot^26^ database using BLASTX program
  The transcripts were matched with Uniprot database using BLASTX^27,28^ program. 70,647 transcripts could successfully find their corresponding homologs from the Uniprot Db. Transcripts that could establish a homology relationship, with E-value <  = 10^−3^ and similarity score >  = 40% were retained in the annotation pipeline for further annotation whereas all others remained un-annotated. The BLASTX profile summary is provided in Table 3. The E-value and similarity-score distribution of BLASTX hits is provided in Fig. 2a,b.

**Table 2:** Assembled transcripts summary.

Number of assembled transcripts	482,871
Number of transcripts after TransDecoder filtering	348,764
Longest transcript length (bp)	44,554
Mean GC % of transcripts	41.60

**Table 3:** Gene Ontology (GO) terms identified in each category using KEGG annotation.

Biological Processes (BP)	5292
Cellular Components (CC)	990
Molecular Functions (MF)	2178

**Fig. 3:**
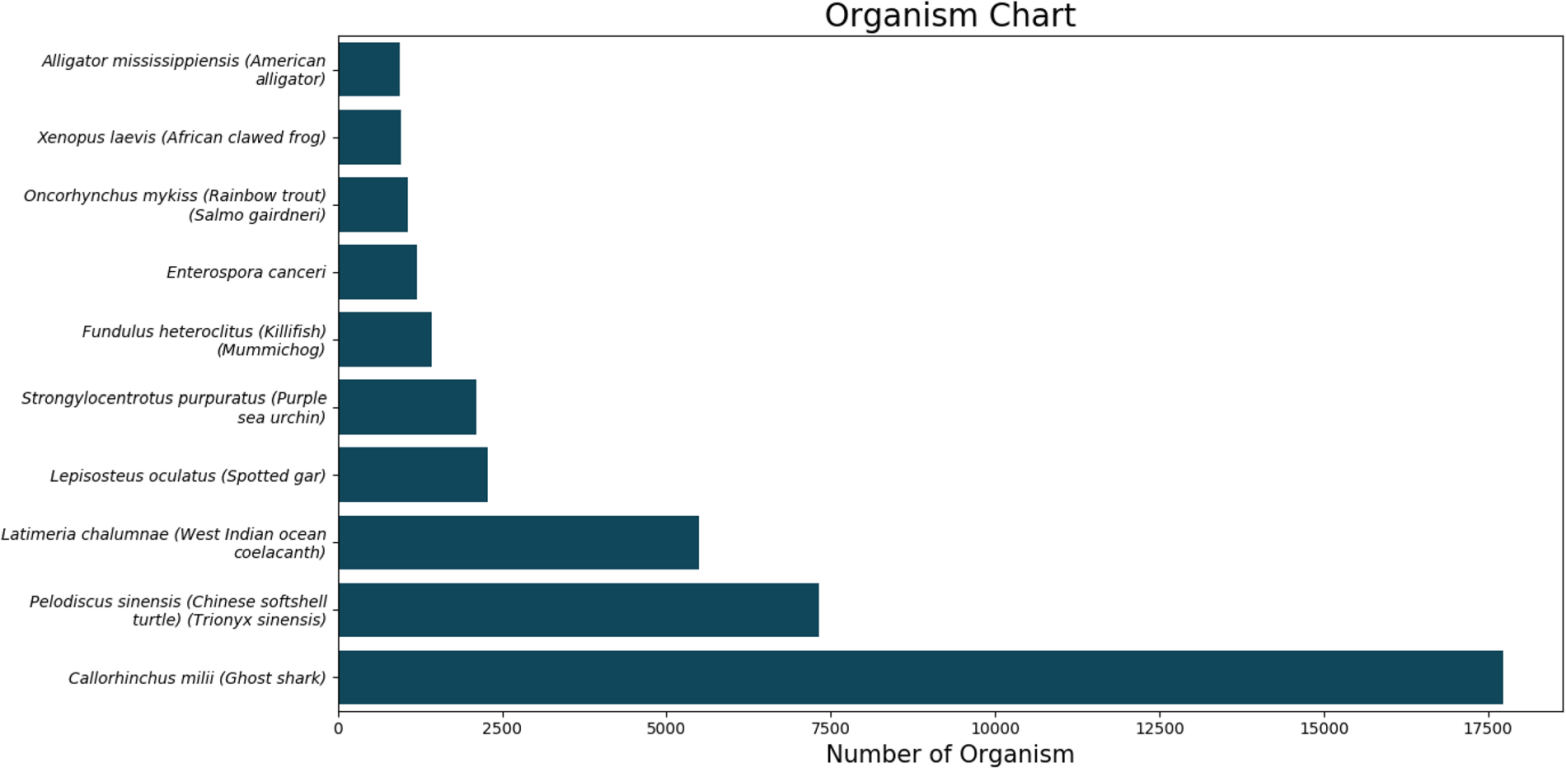
The top 10 BLASTX hits of each transcript after organism annotation.

**Fig. 4:**
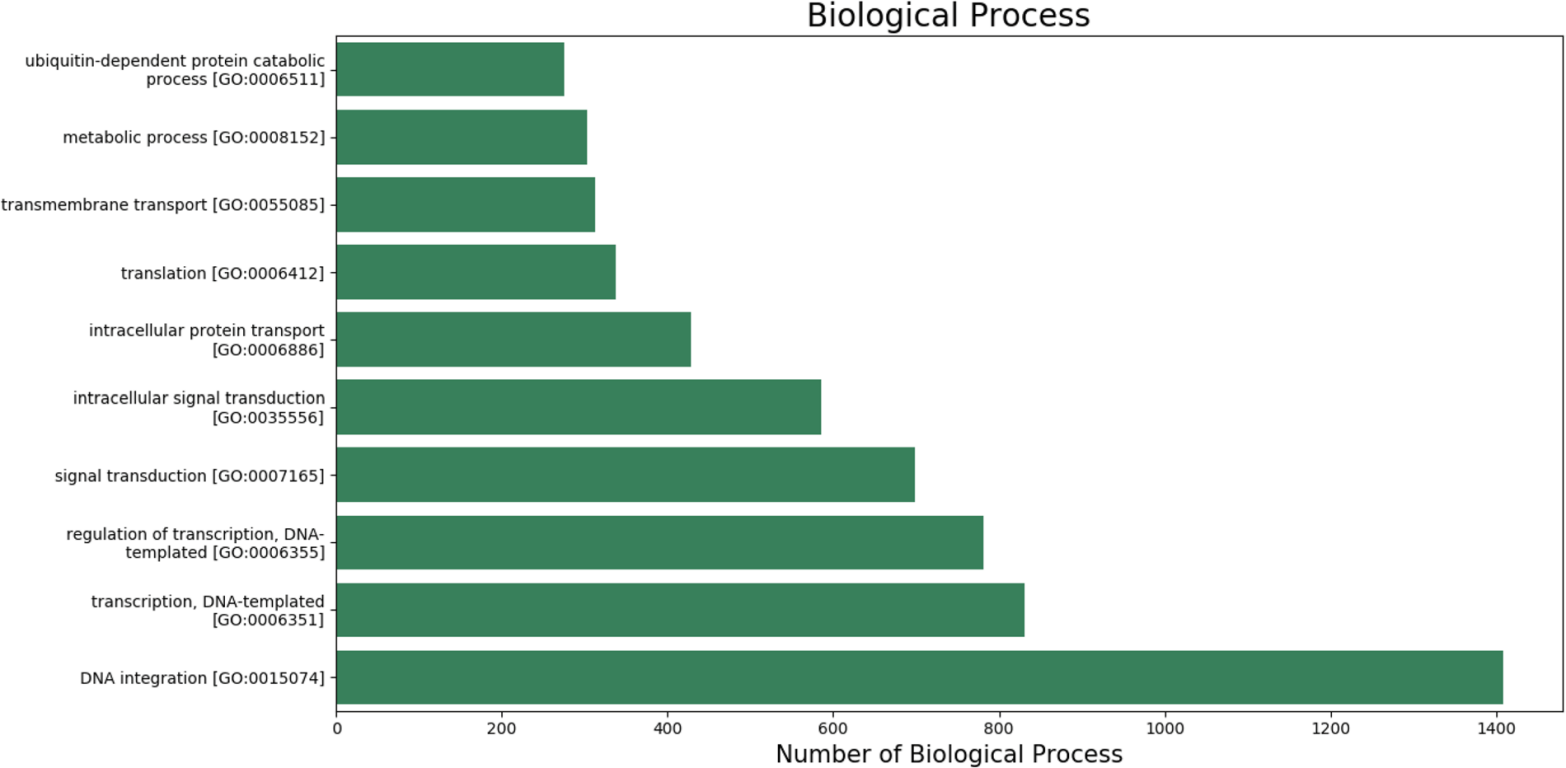
The top 10 GO annotated terms corresponding to ‘Biological Processes (BP)’.

**Fig. 5:**
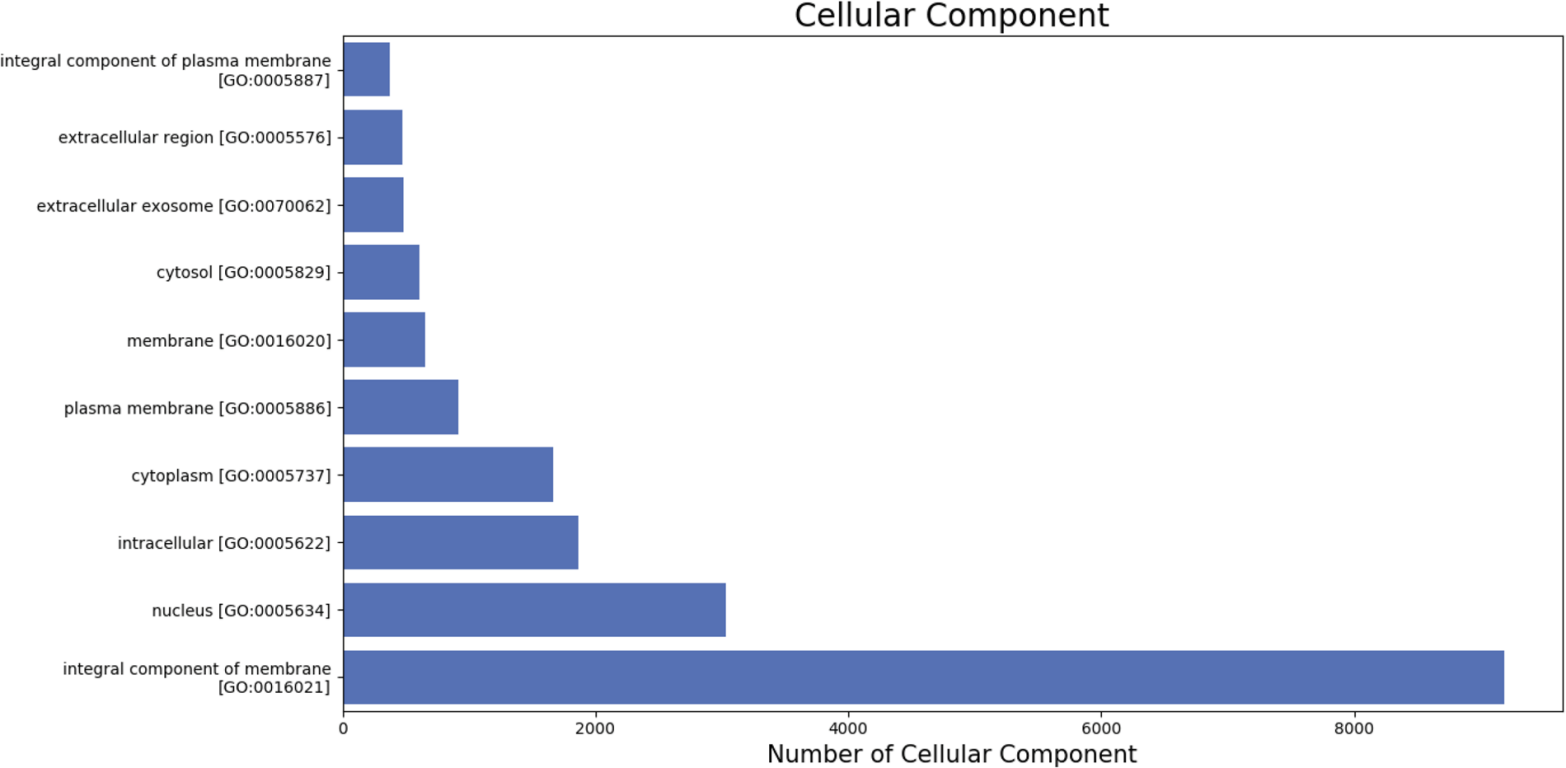
The top 10 GO annotated terms corresponding to ‘Cellular Components (CC)’.

**Fig. 6:**
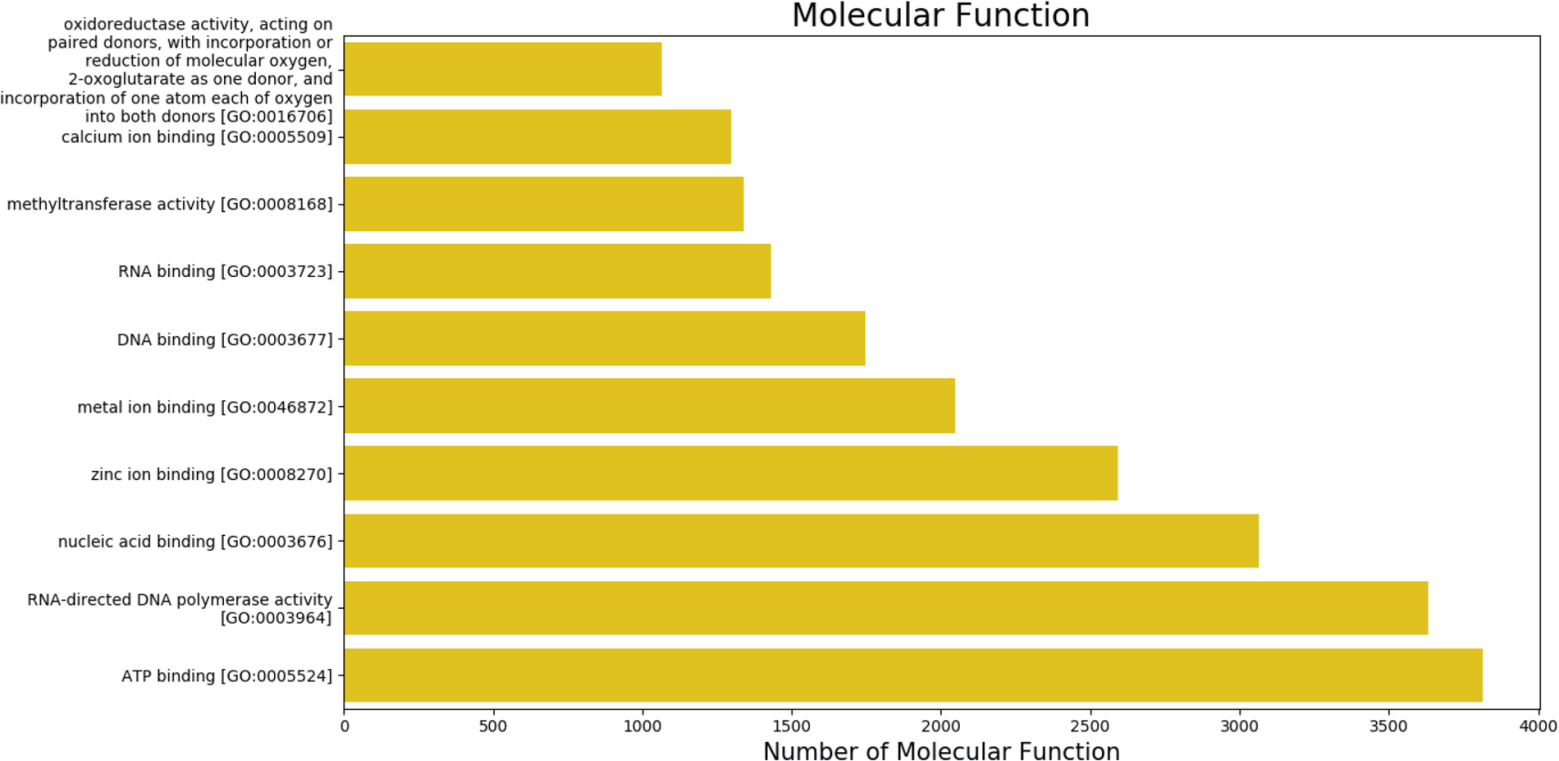
The top 10 GO annotated terms corresponding to ‘Molecular Functions (MF)’.

- Organism annotation
  The top BLASTX hit of each transcript and the organism’s name was extracted. The top10 organisms are displayed in Fig. 3. We further predicted long open reading frames (ORFs) and amino acid sequences using a TransDecoder software (version 5.3.0).

- Gene ontology
  The gene ontology (GO) terms for all the assembled transcripts were extracted wherever possible. The total number of different GO terms identified in molecular function, biological process and cellular component category using KEGG^29^ annotation tool are provided in Table 3. The graphical representation corresponding to biological process (BP), cellular component (cc) and molecular function (mf) is shown in Figs. 4–6.  Also, the final annotated transcriptome assembly is shared on Figshare.

## Data Records

The high-quality sequence data which is free from vector contamination was deposited in the NCBI Sequence Read Archive^30^. The highly curated transcriptome assembly was deposited at DDBJ/EMBL/GenBank through registration to GenBank^31^. The predicted amino acid sequences after TransDecoder filtering, annotated transcriptome assembly, Gene Ontology (GO) and organism annotation outputs, BUSCO results and all the figures are made accessible on Figshare^32^.

## Technical Validation

Trimmomatic^33^ with modified parameters that the Trinity uses (ILLUMINACLIP:$TRIMMOMATIC_DIR/adapters/TruSeq 3-PE.fa:2:30:10 SLIDINGWINDOW:4:5LEADING:5 TRAILING:5 MINLEN:25) was used for the final curation of the trimmed reads. FASTA statistics of the curated assembly is shown in Table 4. Also, the completedness of translated assemblies was further assessed by exploiting the BUSCO (version 5.4.6) platform of the galaxy web server. BUSCO was run in the mode ‘eukaryotic transcriptome’(euk_tran). The output of BUSCO completeness evaluation program generated high scored translated assembly with the vertebrate gene dataset^28^ which is 91.5%. Single copy BUSCOs and duplicated copy BUSCOs contribute to 57.8% and 33.7% of the complete BUSCOs. Fragmented BUSCOs were totally absent and missing BUSCOs with 8.5% of the total coverage. BUSCO was run in the Transcriptome mode generating 3354 BUSCOs of which 3069 were complete BUSCOs, 285 missing BUSCOs, 0 fragmented BUSCOs. Out of the 3069 complete BUSCOs, 1939 single-copy BUSCOs and 1130 duplicated BUSCOs were generated. The complete BUSCO scores computed with the vertebrate gene set are reported in Table 5.

**Table 4:** FASTA statistics of the assembly.

Library	parameter	contigs	Minimum length	Maximum length	Mean length	n50	gc content	Data accession
Pooled reads from female adult liver, heart, spleen, kidney and brain	trimmomatic	348748	200	44554	847	1569	41.60%	GJPK00000000.1^31^

**Table 5:** Completeness assessment of transcriptome assembly using BUSCO.

	Complete (C) BUSCOs		Fragmented (F) BUSCOs	Missing(M) BUSCOs	percentage
BUSCOV5.4.6 + vertebrates (3069 core genes)	Single-copy BUSCOs (S)	1939	0	285	91.50
	Duplicated BUSCOs (D)	1130			

The draft transcriptome assembly of *Chiloscyllium griseum* generated represents a catalogue of gene sets and could therefore be used for gene mining of particular interest. Genes with a characteristic protein coding function, deciphered as ‘immunity’ or ‘stress’ related genes (PCGs), find application in the biomedical field opening up new avenues in the discovery of bio-markers and comparative sequence analysis studies.

## Data Availability

No custom code was generated.

## References

[1] Bright, C. Invasive species: pathogens of globalization. *Foreign Policy*. 50–64 (1999).

[2] Bozzano A, Collin SP (2000). Retinal ganglion cell topography in elasmobranchs. Brain Behav. Evol..

[3] Martin AP, Naylor GJP, Palumbi SR (1992). Rates of mitochondrial DNA evolution in sharks are slow compared with mammals. Nature..

[4] Klimley, A. P. *The biology of sharks and rays* (Chicago Univ. Press, 2013).

[5] Hedges SB (2002). The origin and evolution of model organisms. Nat. Rev. Genet..

[6] Venkatesh B (2006). Ancient noncoding elements conserved in the human genome. Science..

[7] Lee AP, Kerk SY, Tan YY, Brenner S, Venkatesh B (2011). Ancient vertebrate conserved noncoding elements have been evolving rapidly in teleost fishes. Mol. Biol. Evol..

[8] Onimaru K (2015). A shift in anterior–posterior positional information underlies the fin-to-limb evolution. Elife..

[9] Venkatesh B (2014). Elephant shark genome provides unique insights into gnathostome evolution. Nature..

[10] Renz AJ, Meyer A, Kuraku S (2013). Revealing less derived nature of cartilaginous fish genomes with their evolutionary time scale inferred with nuclear genes. PLOS ONE.

[11] Marra NJ (2019). White shark genome reveals ancient elasmobranch adaptations associated with wound healing and the maintenance of genome stability. Proc. Natl. Acad. Sci. USA.

[12] Dlugosch KM, Lai Z, Bonin A, Hierro J, Rieseberg LH (2013). Allele Identification for Transcriptome-Based Population Genomics in the Invasive Plant Centaurea solstitialis. G3 Genes|Genomes|Genetics.

[13] Gayral, P. et al. Reference-free population genomics from next-generation transcriptome data and the vertebrate invertebrate gap. *PLOS GENET*. **9**. 10.1371/journal.pgen.1003457 (2013). 10.1371/journal.pgen.1003457 PMC3623758 23593039

[14] Isaac, N. J. B., Turvey, S. T., Collen, B., Waterman, C. & Baillie, J. E. M. Mammals on the EDGE: Conservation priorities based on threat and phylogeny. *PLOS ONE* **2**. 10.1371/journal.pone.0000296 (2007). 10.1371/journal.pone.0000296 PMC1808424 17375184

[15] Tonini JFR, Beard KH, Ferreira RB, Jetz W, Pyron RA (2016). Fully-sampled phylogenies of squamates reveal evolutionary patterns in threat status. Biol. Conserv..

[16] Lewin HA (2018). Earth BioGenome Project: Sequencing life for the future of life. Proc. Natl. Acad. Sci. USA.

[17] Rhie A (2021). Towards complete and error-free genome assemblies of all vertebrate species. Nature..

[18] Fan G (2020). Initial data release and announcement of the 10,000 Fish Genomes Project (Fish10K). GigaScience.

[19] Nishimura, O. *et al*. Squalomix: shark and ray genome analysis consortium and its data sharing platform. *F1000research* **11**, 1077 (2022). 10.12688/f1000research.123591.1 PMC9561540 36262334

[20] Ebert, D. A., Fowler, S., Compagno, L. & Dando, M. *Sharks of the world*. Wild Nature Press, (2013).

[21] Stein RW (2018). Global priorities for conserving the evolutionary history of sharks, rays and chimaeras. Nat. Ecol. Evol..

[22] VanderWright, W. J. *et al*. *Chiloscyllium griseum*. *The IUCN Red List of Threatened Species* 2020: e.T41792A124416752. 10.2305/IUCN.UK.2020-3.RLTS.T41792A124416752.en. Accessed on 06 August 2023 (2020).

[23] DeTolla LJ (1995). Guidelines for the Care and Use of Fish in Research. ILAR J..

[24] Grabherr MG (2011). Full-length transcriptome assembly from RNA-Seq data without a reference genome. Nat. Biotechnol..

[25] Haas BJ (2013). *De novo* transcript sequence reconstruction from RNA-seq using the Trinity platform for reference generation and analysis. Nat. Protoc..

[26] Pundir S, Martin MJ, O’Donovan C (2017). UniProt Protein Knowledgebase. Methods Mol. Biol..

[27] Altschul SF, Gish W, Miller W, Myers EW, Lipman DJ (1990). Basic. local alignment search tool. J. Mol. Biol..

[28] Zhang Z, Schwartz S, Wagner L, Miller W (2000). A greedy algorithm for aligning DNA sequences. J. Comput. Biol..

[29] Kanehisa M, Goto S (2000). KEGG: Kyoto Encyclopedia of Genes and Genomes. Nucleic Acids Res..

[30] (2022). NCBI Sequence Read Archive.

[31] (2022). NCBI GenBank.

[32] Harshan P, Sukumaran S, Achamveetil G (2023). Figshare.

[33] Bolger, A. M., Lohse, M. & Usadel, B. Trimmomatic: A flexible trimmer for Illumina sequence data. *Bioinformatics* **30**, 2114–2120. 01510 (2014). 10.1093/bioinformatics/btu170 PMC4103590 24695404

